# *In situ* electrosynthesis of quinone-based redox-active molecules coupling with high-purity hydrogen production†

**DOI:** 10.1039/d4sc03033h

**Published:** 2024-07-02

**Authors:** Hyunjoon Ji, Ziming Zhao, Changkun Zhang, Xianfeng Li

**Affiliations:** a Dalian Institute of Chemical Physics, Chinese Academy of Sciences Zhongshan Road 457 Dalian 116023 P. R. China zhangchk17@dicp.ac.cn lixianfeng@dicp.ac.cn; b University of Chinese Academy of Sciences Beijing 100049 P. R. China

## Abstract

Clean hydrogen production *via* conventional water splitting involves sluggish anodic oxygen evolution, which can be replaced with more valuable electrosynthesis reactions. Here, we propose one novel strategy for coupling *in situ* organic electrosynthesis with high-purity hydrogen production. A benzoquinone-derivative disodium 4,5-dihydroxy-1,3-benzenedisulfonate (Tiron)-o1 and a naphthoquinone-derivative 2,6,8-trismethylaminemethylene-3,5-dihydroxy-1,4-naphthoquinone (TANQ) were *in situ* electrosynthesized and directly used in a flow battery without any further purification treatment. Constant, simultaneous production of TANQ and hydrogen was demonstrated for 61 hours, while stable charge–discharge capacities were retained for 1000 cycles. The work provided a new avenue for achieving *in situ* redox-active molecule synthesis and high-purity hydrogen.

## Abstract 2

The asymmetric electrolysis cell concept was introduced to simultaneously produce quinone-based redox-active molecules and high-purity hydrogen gas.

## Graphical abstract

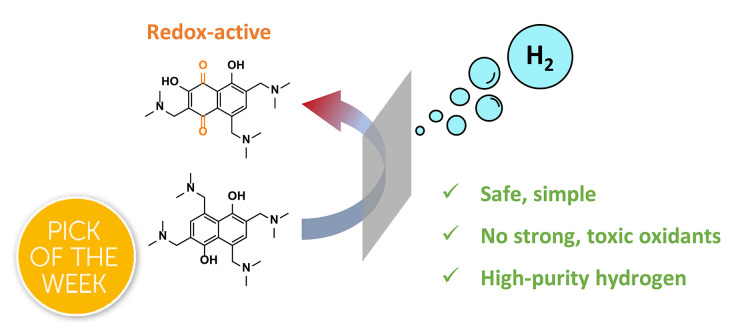

Green hydrogen production by water electrolysis coupled with renewables is getting attention nowadays due to its clean and zero-carbon process.^1^ Optimizing the cost and efficiency of electrolyzers is essential to reaching large-scale production.^2–4^ Conventional water electrolysis involves a hydrogen evolution reaction (HER) coupled with an oxygen evolution reaction (OER). However, the safety and gas purity issues of producing hydrogen and oxygen in a single cell challenge its practical wide application. On the other hand, the sluggish OER always requires high loadings of noble metal catalysts, which increases the cell voltage and cost of the whole electrolysis system. Thus, decoupling HER and OER separately has recently become an important research area.^5–7^

Decoupled water electrolysis, first proposed by Symes and Cronin, requires an intermediate redox mediator circulating between an HER and an OER cell, thus separating them in time or space. Various redox mediators have been proposed, ranging from inorganic metals (V, Ce, Fe, phosphomolybdic acid, phosphotungstic acid, silicotungstic acid)^8,9^ to organic molecules (anthraquinone-2,7-disulfonate, 7,8-dihydroxy-2-phenazinesulfonic acid, tetramercaptopropanesulfonate quinone).^10–14^ The decoupling concept can be further simplified by replacing the bottleneck anodic OER with alternative organic molecule oxidation,^15–17^ including urea, hydrazine, alcohol, aldehyde, amine, and furfural.^18–22^ Quinolines or sulfides were also synthesized for pharmaceutical applications.^23,24^ The HER-coupled electrosynthesis concept improved energy utilization by simultaneously producing high-purity hydrogen with value-added organic molecule synthesis.

Recently, the concept was intensively applied in membrane-divided or undivided (membrane-free) flow cells,^21,25–27^ where divided flow cells prevent the mixing of hydrogen and oxygen and maintain high purity of hydrogen gas. Various metal-based catalysts have also been applied (Ni, Co, Mo, Cu) to control synthesis yield and selectivity.^28,29^ However, using such catalysts on an industrial scale would require additional efforts and increase the overall cost. The research still requires optimization for safe, simple, and inexpensive electrolysis. Molecules with redox activity have recently been widely investigated as redox species for organic flow batteries (OFBs).^30–36^ However, the preparation of these organic redox-active molecules (ORAMs) always involves strong, toxic solvents, high-cost catalysts, and additional purification steps to optimize their solubility, stability, and redox potential.^37^

In this work, we propose one effective strategy for quinone-based ORAMs' *in situ* electrochemical synthesis coupling with continuous high-purity hydrogen production. The new asymmetric electrolysis cell concept enabled the simultaneous production of ORAMs and high-purity hydrogen gas (Fig. S1†). Constant hydrogen production for 61 hours was also demonstrated. Compared to chemical oxidation, the *in situ* electrosynthesis enabled a safe and easy route under relatively mild conditions. Meanwhile, the synthesized ORAMs can be used in OFBs directly without any further purification treatment. The flow battery (FB) experiment also displayed a high coulombic efficiency of 100% and energy efficiency of 70% at 40 mA cm^−2^ for 1000 cycles. Instead of producing simple sacrificial or low-value chemicals, this work introduced a novel approach to producing electrochemically reversible battery electrolytes and high-purity hydrogen in parallel.

## Results and discussion

**Fig. 1:**
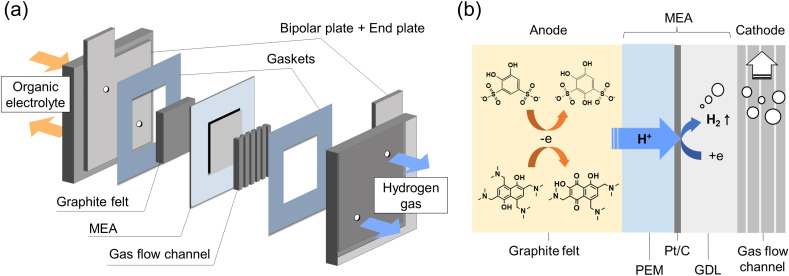
Flow electrolysis cell: (a) schematic diagrams of the flow electrolysis cell; (b) detailed cross-section at MEA.

The schematic diagram of the *in situ* electrolysis cell is displayed in Fig. 1a, in which a membrane electrode assembly (MEA) was placed between the anode and cathode. The MEA was prepared by spraying a Pt/C (28 wt%) suspension onto the Nafion 212 membrane as the HER catalyst (Fig. S2†) The Pt loading was around 0.16 mg cm^−2^. On the anode side, commercial carbon felt was used with no additional treatment. On the cathode side, a gas diffusion layer (carbon paper) and a gas flow channel were tightly assembled with MEA to facilitate hydrogen gas transport. Constant current was given during the electrolysis, where the protons from the organic electrolyte were transferred to the cathode through MEA by electro-osmotic drag and then catalyzed by Pt/C generating hydrogen (Fig. 1b). Meanwhile, organic molecules were constantly synthesized at the anode.

**Fig. 2:**
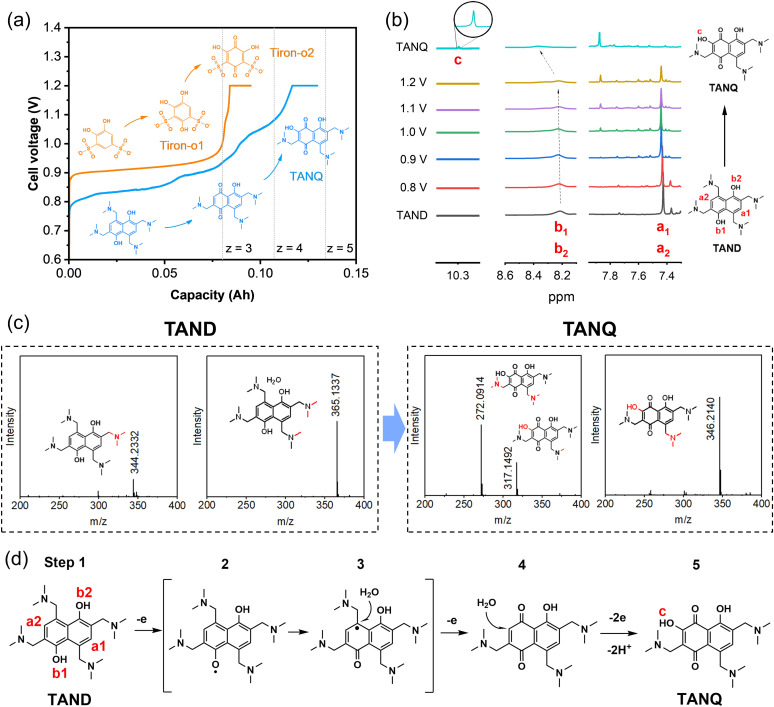
*In situ* electrosynthesis of TANQ: (a) cell voltage during *in situ* electrosynthesis of TANQ (blue) compared with Tiron-o1 (orange). Dotted lines (*z* = 3, 4, 5) represent the accessible theoretical capacity according to the number of electrons (*z*) transferred, (b) *ex situ*^1^H NMR spectra taken at different cell voltages, (c) LC-MS spectra before and after electrosynthesis (detached parts during ion excitation in the ion source are shown in red and the specific position was not identified). (d) Proposed route of TANQ electrosynthesis.

Tiron (disodium 4,5-dihydroxy-1,3-benzenedisulfonate), a benzene ring with two pairs of hydroxyl and sulfonic groups, was selected as one precursor for molecule synthesis. As shown in Fig. 2a, the cell voltage reached around 0.9 V at the constant current density of 10 mA cm^−2^ at room temperature for 0.1 M Tiron in 1 M H_2_SO_4_ (orange line), consistent with the onset potentials in cyclic voltammetry measurements and cell polarization curve (Fig. S3†). The long voltage plateau between 0.9 V and 1.0 V can be attributed to the period where most Tiron molecules were substituted with an additional hydroxyl group. The molecular structure change was measured by *ex situ*^1^H NMR at every 24% capacity interval (Fig. S4a†). At 0% SOC, two aromatic protons were observed (doublets at 7.20 and 7.44 ppm, represented as *a* and *b*). The intensity of the two signals gradually decreased and finally disappeared during oxidation. The ring was slowly occupied by nucleophilic water addition, attaching a new hydroxyl group at the 4-position of carbonyl (position b), which we named Tiron-o1. Meanwhile, a new singlet at upfield 7.14 ppm constantly grew until the end of oxidation, implying that another aromatic proton (position a) remained unsubstituted. A short voltage plateau between 1.0 V and 1.2 V was also detected in Fig. 2a, which reflected the further conversion to two-hydroxyl substitution (Tiron-o2). The final product was a mixture of Tiron-o1 and Tiron-o2 (Fig. S4b and Table S1†), according to liquid chromatography-mass spectrometry (LC-MS) analysis. Only a little amount of the initial Tiron was detected in the final product, evidencing high conversion ratio.

Meanwhile, a constant hydrogen production rate of 0.3 ml min^−1^ was measured during constant current 10 mA cm^−2^. The cell outlet was directly connected to gas chromatography (Fig. S5†). No molecule crossover was detected from the anode to the cathode during the electrolysis, confirming high-purity hydrogen production. A total of 40 ml of hydrogen was collected during the whole electrolysis (Experimental section in the ESI†). Since the oxidation from Tiron to Tiron-o2 requires 6 electrons theoretically(Fig. S4c†),^38^ our electrolysis reached 59% to achieve mainly Tiron-o1 and partially Tiron-o2, corresponding to the cell charge voltage plateau and the area ratio of 2(Tiron-o1) : 1(Tiron-o2) in the LC-MS analysis.

After electrolysis, the *in situ* synthesized molecule was transferred to a flow cell for long-term cycling against V^2+^/V^3+^ as the anode redox couple. Cycling showed an average coulombic efficiency of 100%, while energy and voltage efficiencies were above 70% at the current density of 40 mA cm^−2^. However, the cell quickly underwent capacity decay and reduction in open circuit voltage (Fig. S6a†), which could be attributed to Michael attack and the instability of the synthesized molecule.^38,39^ Moreover, charge–discharge curves exhibited up to three plateaus, meaning at least two reversible transformations continued during battery cycling (Fig. S6b†). Therefore, synthesizing an ORAM with a more robust, irreversible transformation and long-term stability was necessary.

To improve the ORAM stability, we then synthesized the naphthalene derivative 2,6,8-trismethylaminemethylene-3,5-dihydroxy-1,4-naphthoquinone (TANQ) *via* this *in situ* electrochemical method. Both Tiron-o1 and TANQ were prepared from redox-inactive benzene- or naphthalene-derivatives, which became electrochemically reversible quinone structures after *in situ* electrosynthesis. Its precursor 2,4,6,8-tetramethylaminemethylene-1,5-naphthalenediol (TAND) was obtained from 1,5-dihydroxynaphthalene with four alkylamines by Mannich reaction (Experimental section in the ESI†). The cell voltage of the *in situ* electrochemical synthesis started from around 0.8 V and went through at least three different plateaus until reaching 1.2 V, indicating several structural changes in the molecule (Fig. 2a, blue line). The average cell voltage was lower compared to that of Tiron, which can be attributed to the properties of molecules, such as the intrinsic molecular structure and the number, position, and types of functional groups.^40^ The *ex situ*^1^H NMR samples were taken at different stages as shown in Fig. 2b. Peaks of symmetric aromatic protons at a1 and a2 (7.4 ppm) slowly disappeared due to breakage of structural symmetry, followed by the simultaneous formation of a new peak around 7.9 ppm. Peaks also grew at around 7.3 ppm and 8.1 ppm, which can be attributed to different positions of protons existing at the same time. Possible structures are given in Table S2.† Electron loss and delocalization at hydroxyl groups b1 and b2 (gradual shift and fade around 8.3 ppm, represented as intermediate step 2 in Fig. 2d) were followed by water attachment (intermediate step 3), resulting in a quinone form (step 4). A chemical shift of 10.3 ppm (signal c) was observed after the attachment of a hydroxyl group, which could be attributed to the deshielding in the electron-deficient structure of TANQ (step 5). The final product showed no more signal at 7.4 ppm, evidencing complete conversion of the original TAND. LC-MS analysis also showed the structure of TANQ (Table S2†). Analysis using infrared- and Raman spectroscopy showed no obvious structural change. Total 57 ml of hydrogen gas was collected at the cathode (98% of the capacity at 130 mA h), which was the equivalent of 4.84 electrons transferred, somewhat higher than our estimation *z* = 4. Such discrepancy can be attributed to additional electron consumption to produce molecules other than TANQ (blue-shaded in Table S2†), although the portion was relatively small.

**Fig. 3:**
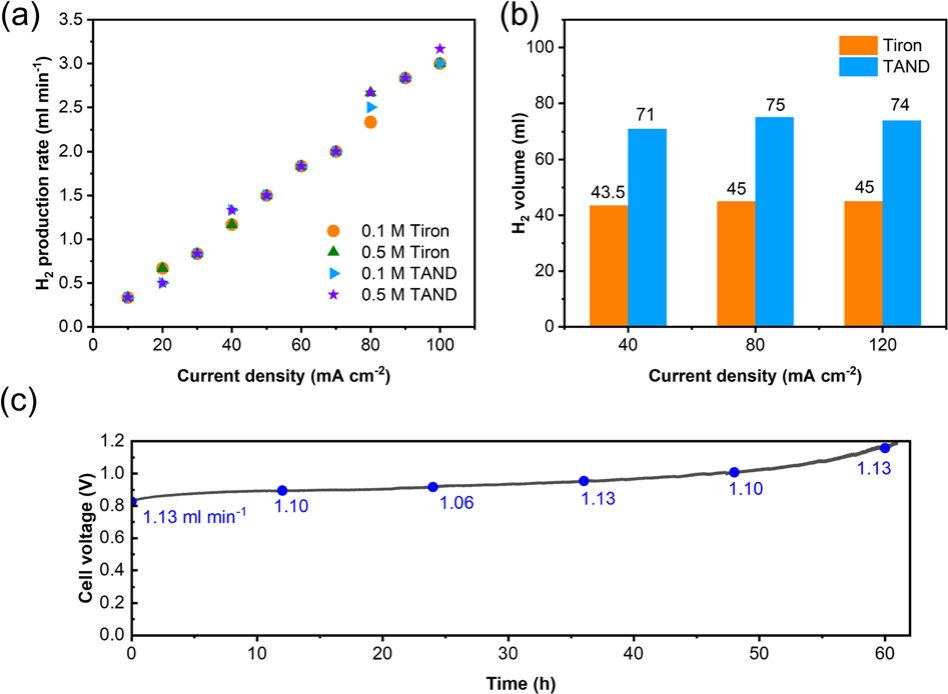
Cell characterization at different current densities and concentrations. (a) Hydrogen production rate during stepwise-LSV at concentrations 0.1 M/0.5 M. (b) Collected hydrogen volume in higher current densities using 10 ml of 0.1 M Tiron/TAND in 1 M H_2_SO_4_. (c) Performance of long-term electrolysis using 2 liters of 0.1 M TAND electrolyzed at 40 mA cm^−2^. Gas production rates (ml min^−1^) are depicted every 12 hours.

High-purity hydrogen was also constantly produced in the cathode during TANQ electrosynthesis (Fig. S5†). Gas production rates (ml min^−1^) at higher current densities and molecule concentrations were measured by 3 minutes-step linear sweep voltammetry (Fig. S7†) at cell temperature 60 °C. Both Tiron-o1 and TANQ retained the same redox activity at a higher temperature (Fig. S8†). The hydrogen production rate of 1.33 ml min^−1^ was measured at 40 mA cm^−2^, comparable to other reported values.^26^ The gas production rate was linearly dependent on current densities, regardless of the molecule type or concentration (Fig. 3a). No gas evolution was observed in the anode, evidencing a safe voltage range. Even at much higher current densities (>100 mA cm^−2^), the linearity of the hydrogen production rate was maintained (Fig. S9†), reaching 16.5 ml min^−1^ at 500 mA cm^−2^. The same linear tendency would be expected in the anode organic production. However, current densities were relatively lower than those of pure water splitting, which can be attributed to the slow reaction kinetics of molecules and high membrane resistance at room temperature. Cell polarization can be decreased by using high-activity catalysts, high ion-conductivity membranes, and high flow rates. Electrolysis at different current densities of 40, 80, and 120 mA cm^−2^ verified that similar amounts of hydrogen were produced regardless of the current densities (Fig. 3b). This implied that hydrogen production is always limited to the capacity of the anodic electrosynthesis. Energy consumption of 2.64 kW h m^−3^ H_2_ and 2.58 kW h m^−3^ H_2_ was recorded for TAND and Tiron at 120 mA cm^−2^ respectively, which was relatively low compared to conventional water splitting or HER-coupled biomass electrooxidation.^25^ Experiments at a higher concentration of 0.5 M (Fig. S10c and d†) also showed that electrolyte concentration only extended the total hydrogen volume but did not influence the gas production rate. High cell resistance was inevitable at concentration 1 M TAND, proving the importance of suitable electrolyte concentration. Acid concentrations of 1 M and 3 M were tested using 0.1 M TAND, where the capacity using 3 M acid was slightly higher than that using 1 M acid, possibly due to enhanced solubility and conductivity (Fig. S11†). Electrolysis at a larger scale exhibited long-term stability. 2 Liters of 0.1 M TAND were electrolyzed at a constant current of 40 mA cm^−2^, followed by a constant voltage of 1.2 V (Fig. 3c and S12†). The constant-current electrolysis reached around 61 hours, where a continuous gas production rate of around 1.1 ml min^−1^ was maintained. (Fig. S12†) A total capacity of 26.68 A h was achieved during electrolysis, equivalent of 11.96 Liters of hydrogen gas.

**Fig. 4:**
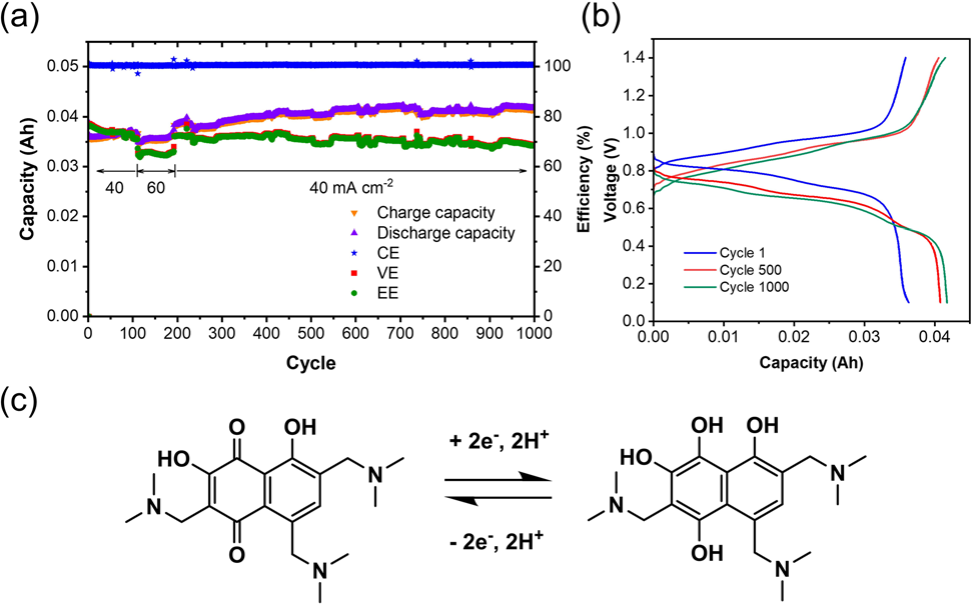
Battery performance of TANQ after electrosynthesis: (a) cycling performance, (b) charge and discharge profiles, (c) structural change of TANQ during charge–discharge cycling. The flow cell was 0.1 M TAND/0.5 M V^2+^ in 3 M H_2_SO_4_ cycled at 40 mA cm^−2^ (cycle 111–192 temporarily operated at 60 mA cm^−2^).

After electrolysis, the synthesized TANQ electrolyte was directly transferred to a FB for long-term cycling evaluation (Fig. 4). The cell exhibited an average coulombic efficiency of 100%, while energy and voltage efficiencies were above 70%. Capacities kept increasing slightly for the first 500 cycles, which is due to the incomplete electrochemical synthesis and the synthetic structural changes continued along cycling. Charge–discharge cycles were characterized by shifting between hydroquinone and quinone with two electron transfers. Two carbonyl groups of TANQ were transformed into hydroxyl groups during discharge, causing a change in electron density coming from the emergence and disappearance of the quinone structure. The fluctuation of the cell capacity and efficiencies during cycling can be attributed to the room temperature change and periodic recharging of V^2+^. The cell capacity was more stable than the Tiron-o1 after 1000 cycles. Our experiment revealed that the *in situ* electrosynthesis strategy is a promising option for ORAM preparation and high-purity hydrogen generation.

## Conclusions

In this work, the *in situ* electrosynthesis of ORAMs coupling hydrogen production was demonstrated. The asymmetric flow electrolysis cell enabled high-purity hydrogen production while simultaneously achieving the electrochemical synthesis of Tiron-o1 and TANQ quinones. The obtained molecules can be directly applied in the FB without additional treatment and TANQ showed more stable capacity retention when compared to the Tiron-o1 molecule after 1000 cycles. Meanwhile, 61 hours of constant hydrogen production rate was achieved by long-term *in situ* electrolysis. Our research expanded the electrosynthesis coupling concept to ORAMs and high-purity hydrogen, which is promising for sustainable energy applications.

## Data availability

All data supporting this article are available as part of the article and its ESI file.†

## Author contributions

Hyunjoon Ji: conceptualization, experimental work, writing the initial draft. Ziming Zhao: discussion of the results. Changkun Zhang and Xianfeng Li: conceptualization, writing – review & revision, supervision. All authors reviewed the manuscript.

## Conflicts of interest

There are no conflicts to declare.

## Supplementary Material

SC-015-D4SC03033H-s001
