# Supplementary material for: In situ electrosynthesis of quinone-based redox-active molecules coupling with high-purity hydrogen production
Source: Chem Sci. 2024 Jul 2;15(33):13185–90. doi: 10.1039/d4sc03033h (PMC11370267; doi:10.1039/d4sc03033h)
Supplement: SC-015-D4SC03033H-s001 [file SC-015-D4SC03033H-s001.pdf]

Electronic Supplementary Material (ESI) for Chemical Science.
This journal is © The Royal Society of Chemistry 2024

Supporting Information

Hyunjoon Ji[a,b], Ziming Zhao[a], Changkun Zhang[a] and Xianfeng Li[a]**

[a] Dalian Institute of Chemical Physics, Chinese Academy of Sciences, Zhongshan Road 457, Dalian 116023, P. R. China.

[b] University of Chinese Academy of Sciences, Beijing 100049, P. R. China

*Corresponding author E-mails: zhangchk17@dicp.ac.cn and lixianfeng@dicp.ac.cn

Experimental

Chemicals

All chemicals were used as received. 1,5-dihydroxynaphthalene was purchased from Beijing Innochem Science & Technology. Formaldehyde was purchased from Liaoning Quan Rui Reagent Co. Ltd. Tiron and dimethylamine (40 %) solution was purchased from Shanghai Titan Technology Co., Ltd.(Adamas-beta®).

Synthesis of TAND

Naphthalene was functionalized with alkylamine groups using Mannich reaction to increase its solubility. 1.6 g 1,5-dihydroxynaphthalene, 50 ml ethanol, 6.25 ml formaldehyde, and 10 ml dimethylamine solution were mixed all together and stirred in a pressure vessel at 80°C for 10 hours. Then the mixture was vacuum-dried at 70°C. The fully dried product was dissolved either in 1 M H_2SO_4 to prepare 0.1 M TAND or in 3 M H_2SO_4 to prepare 0.5 M/1M TAND. For higher concentrations, 0.5 M / 1.0 M TAND and 0.5 M Tiron were dissolved in 3 M H_2SO_4.

Electrochemical methods

Cyclic voltammetry was measured using a three-electrode cell and a Bio-logic VSP 300 potentiostat. A graphite plate with an area 1 cm^2 was used as the working electrode, another graphite plate with an area 4 cm^2 as the counter electrode, and a Hg/Hg_2SO_4 electrode (Tianjin Aida, +0.652 V vs. SHE) as the reference electrode.

Linear sweep voltammograms of the electrolysis cell were acquired using Arbin instrument. Current ramp of 0.005 A s^{-1} was applied until the cell voltage reached either current density 100 mA cm^{-2} or cell voltage 1.4 V. Electrolysis experiments were also conducted using Arbin instrument.

Preparation of membrane electrode assembly (MEA) and electrolysis cell

Membrane electrode assembly (MEA) was prepared as follows: 10 mg of 28 wt.% Pt/C was mixed with 0.5 ml ethanol, 0.5 ml DI water, and 40 µl of 5% Nafion solution. After 1 hour of ultrasonication, the mixture was sprayed on 9 cm^2 of Nafion® 212 membrane on a 60°C hot plate to guarantee a minimum Pt surface density of 0.16 mg cm^{-2}. 0.2 mm gas diffusion layer (Toray, TGP-H-060) was placed on the catalyst layer, and the whole assembly was hot-pressed altogether with sealing frames at 100°C as in Fig S2.

The electrolysis cell was assembled where MEA was sandwiched between anode and cathode. In the anode, a 4 cm^2 (2 x 2 cm, thickness 5 mm) carbon felt was placed. In the cathode, a gas diffusion layer and a gas flow channel were assembled as cells to facilitate hydrogen gas transport. Two graphite bipolar plates and stainless-steel end plates were pressed from both sides for assembly.

Paired electrolysis and hydrogen collection

0.1 M TAND and 0.1 M Tiron were prepared 10 ml each. The cell was charged with a constant current of 10 mA cm^{-2}. After the cell voltage reached a cut-off voltage of 1.2 V, the cell was continuously charged at a constant voltage of 1.2 V until the current density was diminished to 1.25 mA/cm^2. The electrolyte was pumped using a peristaltic pump (YZ1515x, Chonry Peristaltic pump Co., Ltd) at the flow rate 60 ml min^{-1}. The hydrogen gas was collected using an inverted cylinder glass.

Flow battery cycling

The flow battery cell (active area 3 x 3 cm^{-2}) was assembled using carbon felt at both anode and cathode. Two graphite bipolar plates and stainless-steel end plates were pressed from both sides for assembly. 10 ml sample in 3 M H_2SO_4 from the above electrolysis was transferred to the anode. 10 ml of 0.5 M V^{2+} in 3 M H_2SO_4 was prepared as the cathode. The cell was cycled with a constant current of 40 mA cm^{-2} between 1.4 V and 0.1 V for 1000 cycles. During the cycling, 3 M H_2SO_4 was periodically added to the anode to balance the water crossover, while V^{2+} was periodically added to the cathode to prevent complete oxidation.

Material analysis

The product gas was analyzed using gas chromatography (GC-2014, Shimadzu). The electrolysis cell outlet was directly connected to GC to measure the gas product online. Current 10 mA cm^{-2} and 40 mA cm^{-2} were used for electrolysis. 10 ml min^{-1} Ar gas was used as carrier gas. Gas measurement started 10 minutes after starting the electrolysis.

NMR samples were analyzed using AVANCE III 400 MHz (Bruker). 1 ml TAND electrolyte was extracted before and during the electrolysis at cell voltages 0.8, 0.9, 1.0, 1.1, 1.2 V and at fully oxidized state. In the case of Tiron, samples were taken at the state of charge (SOC) 0, 24, 48, 72, 96, and 100%. For TAND, samples were taken when cell voltage reached 0.8, 0.9, 1.0. 1.1, 1.2 V, and before and after the electrolysis. All samples were mixed with D_2O at a volumetric ratio of 8:2.

Mass spectra were measured using liquid chromatography (Q-TOF 6540(Agilent) or Ultimate 3000 UHPLC - Q Exactive (Thermo Scientific)), where the samples were diluted 10 times using water before the measurements.

Supplement Figures

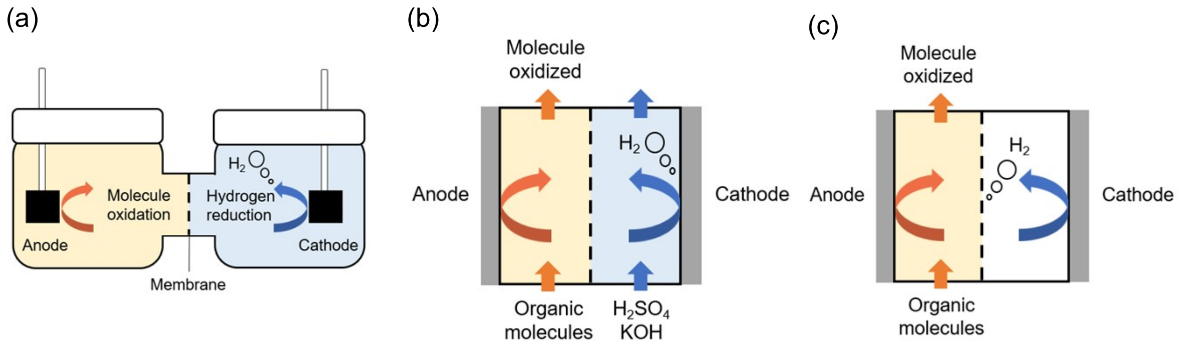

Figure S1. Concepts of divided cells for hybrid electrolysis (a) H-shaped batch type, (b) symmetric flow type (c) asymmetric flow type (This work)

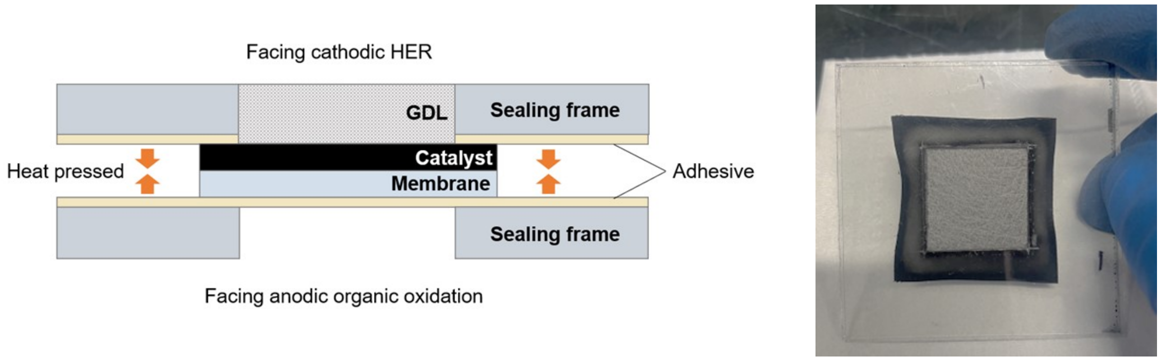

Figure S2 Schematic of the membrane electrode assembly (MEA) (left) and the photograph of the final product (right)

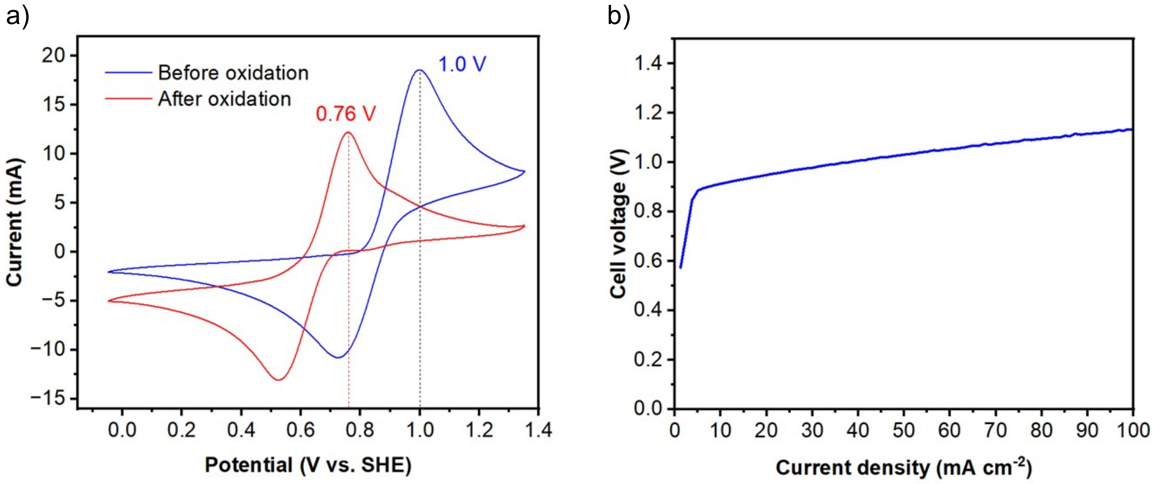

Figure S3 a) Cyclic voltammogram of 0.1 M Tiron before and after electrooxidation and b) polarization curve of the electrolysis cell with anode filled with 0.1 M Tiron

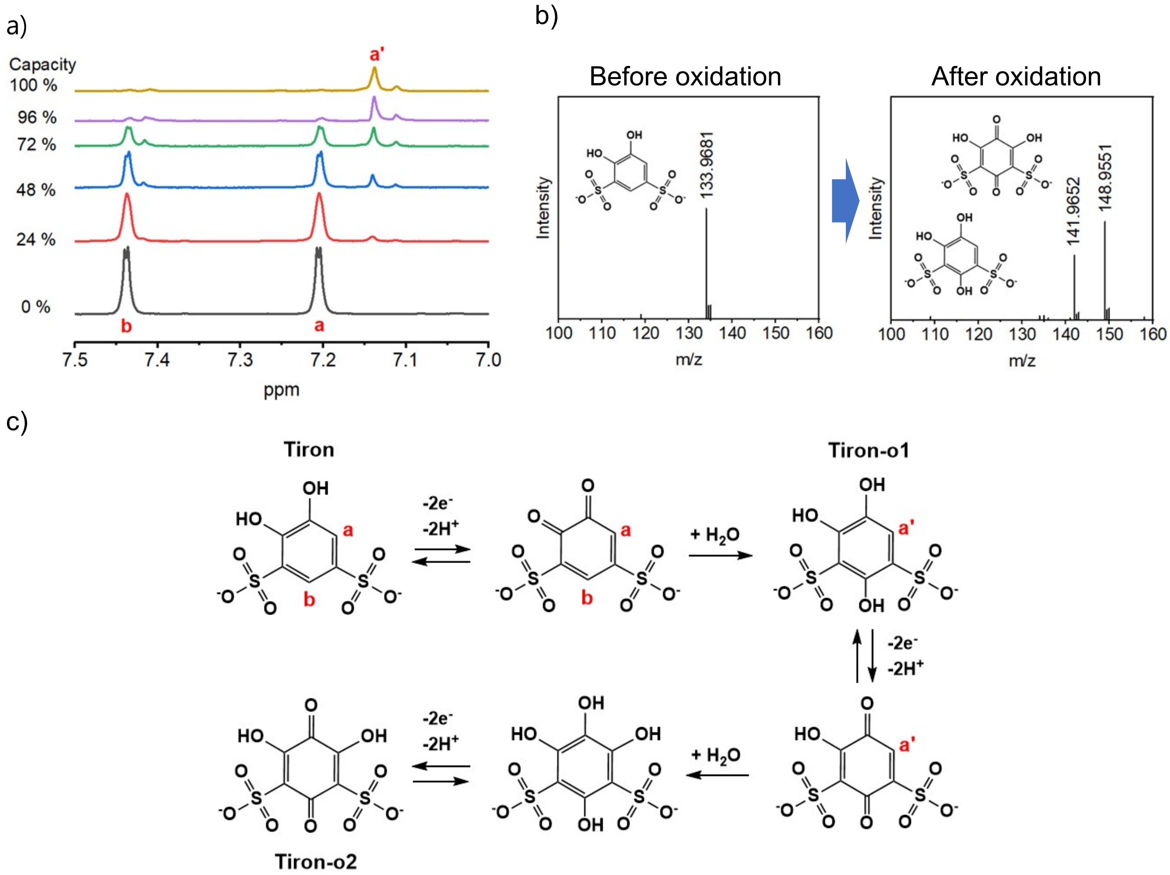

Figure S4 In-situ electrooxidation of Tiron, a) ^{1}H NMR spectra at different SOCs, b) LC-MS mass spectra before and after electrolysis, c) Proposed route of Tiron oxidation[1]

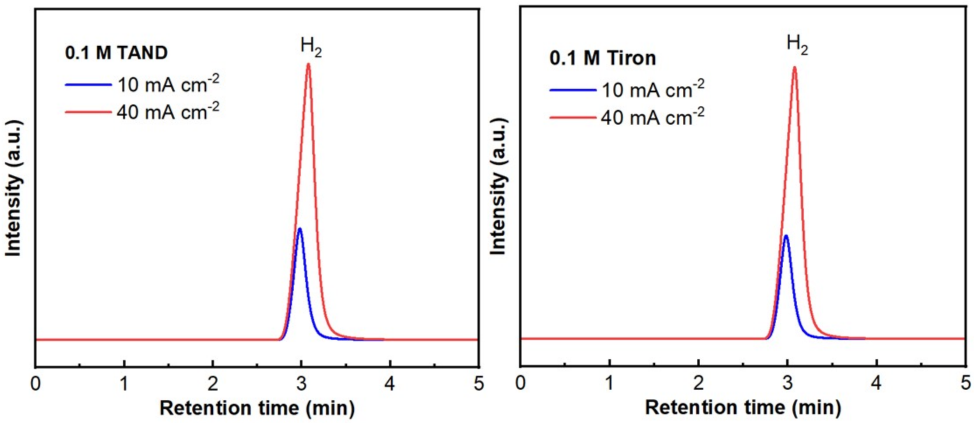

Figure S5 GC measurement of the product gas during Tiron and TAND electrolysis

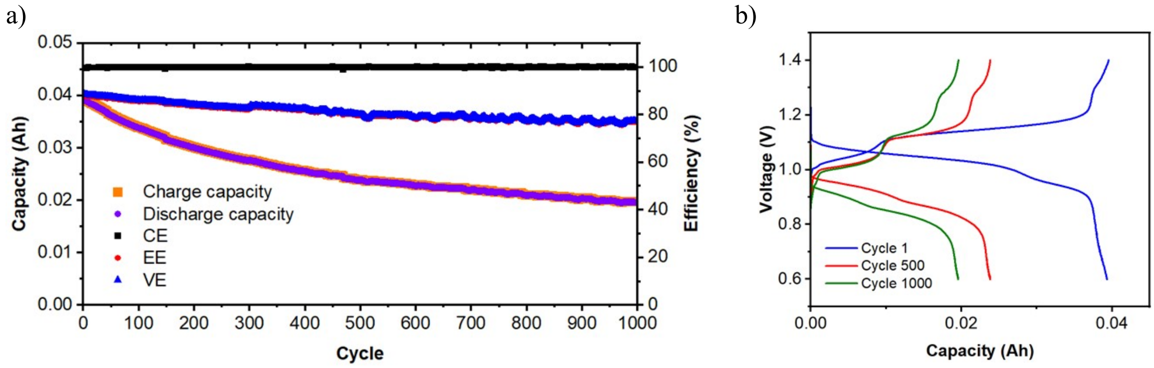

Figure S6 Voltage profile of long-term electrolysis using 10 ml of 0.1 M Tiron at 40 mA cm^{-2}

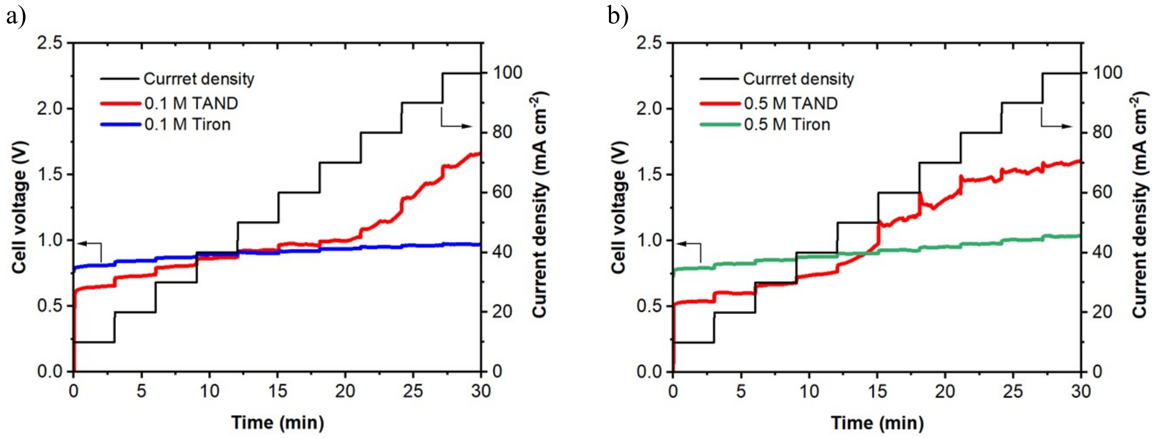

Figure S7 3-minute-steps LSV curves at concentration a) 0.1 M and b) 0.5 M

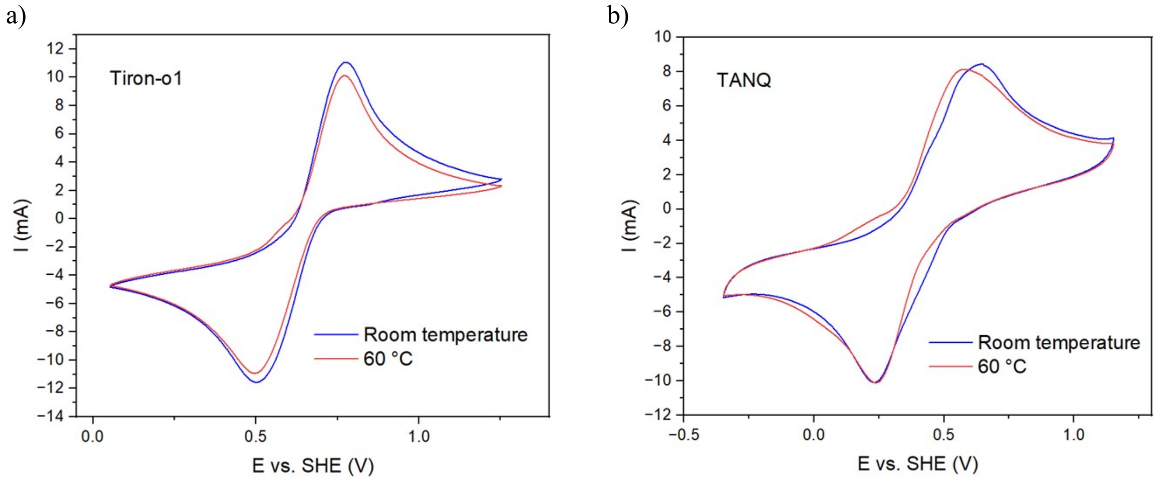

Figure S8 Cyclic voltammogram of (a) Tiron-o1 and (b) TANQ after electrolysis at different temperatures

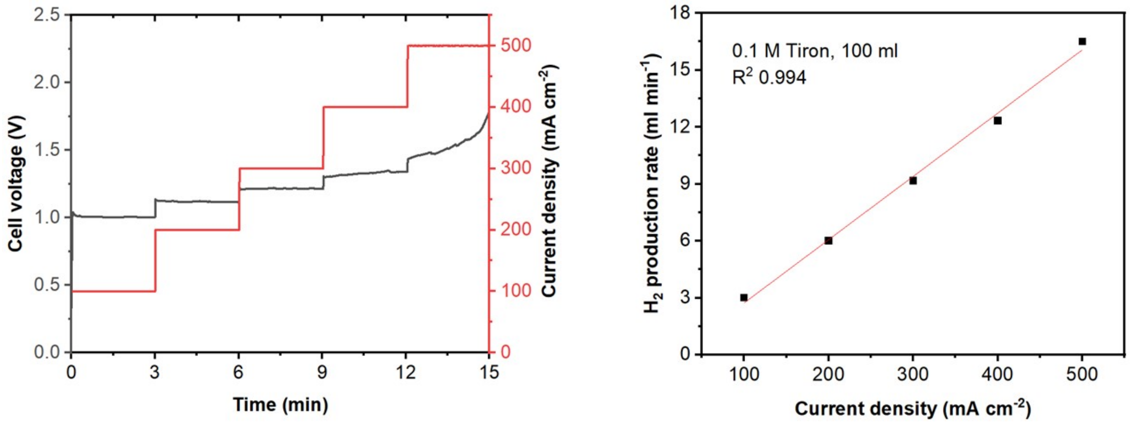

Figure S9 a) 3-minute-steps LSV curve during hybrid electrolysis using 0.1 M Tiron, 100 ml, b) corresponding hydrogen production rates

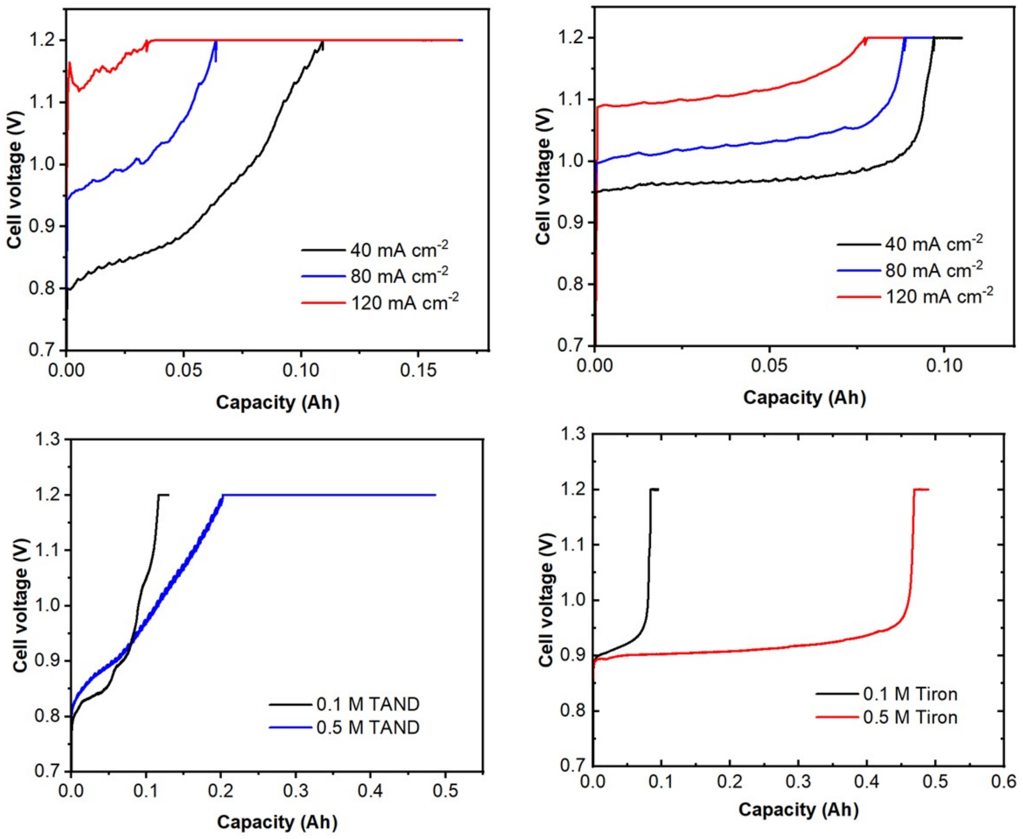

Figure S10 Paired electrolysis in different current densities and concentrations

Voltage profile during electrolysis at different current densities using (a) 0.1 M TAND, (b) 0.1 M Tiron, and (c) 0.5 M TAND, (d) 0.5 M Tiron, both at current 10 mA $cm^{-2.}$ All samples were 10 ml volume.

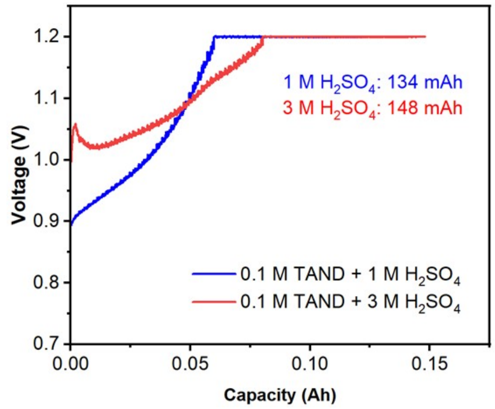

Figure S11 Paired electrolysis in different sulfuric acid concentrations at current 40 mA cm^{-2}

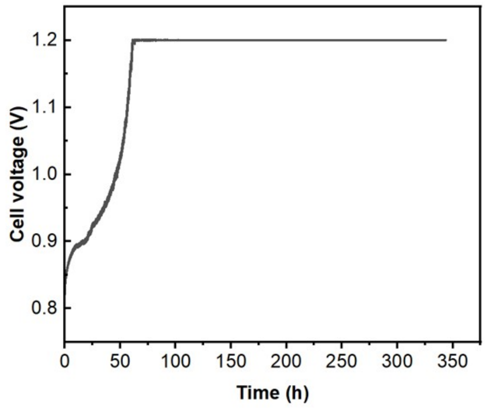

Figure S12 Voltage profile of long-term electrolysis using 2 L of 0.1 M TAND at 40 mA cm^{-2}

Table S1 LC-MS Mass spectra of Tiron samples and estimated molecule structures

Total ion chromatogram (Spectra extracted at blue and red dots)

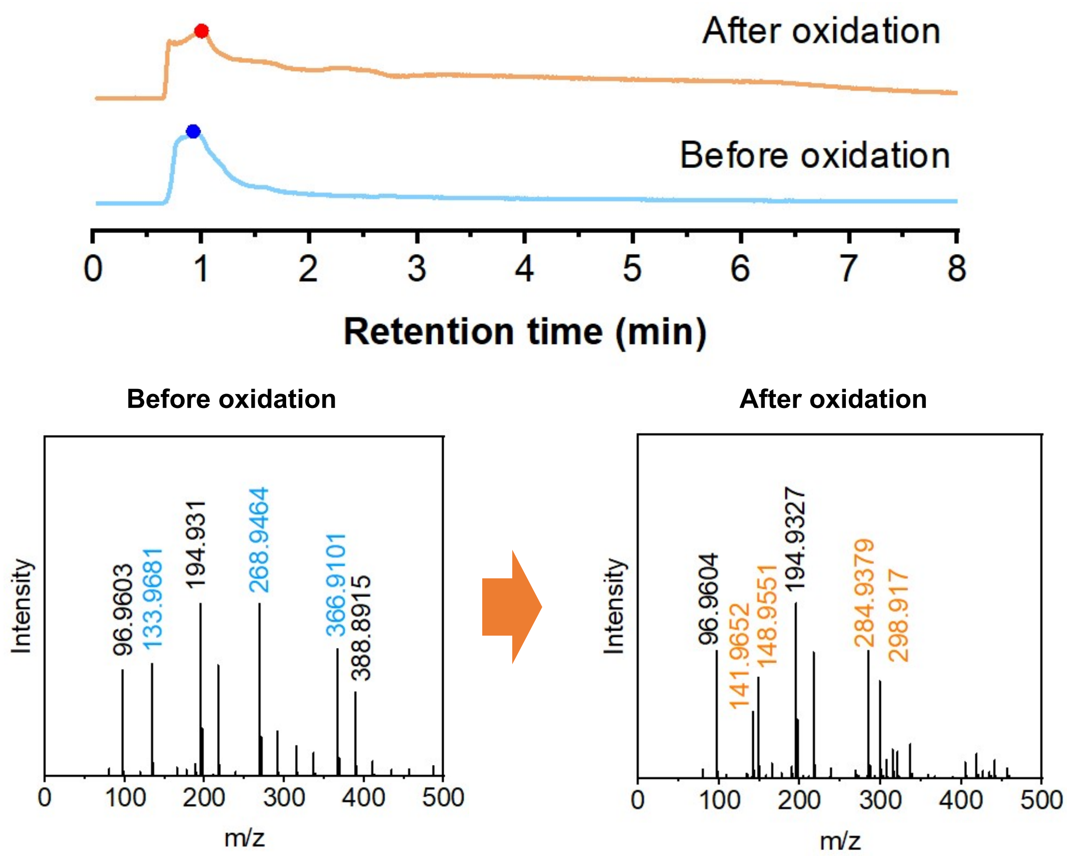

m/z	Estimated structure	m/z	Estimated structure
133.9681/268.9464	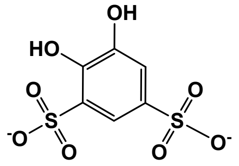 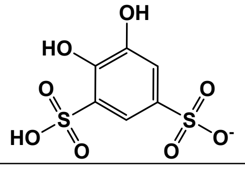	141.9652/284.9379	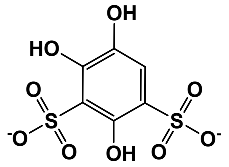 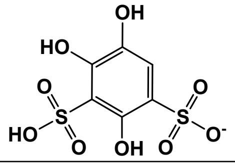
366.9101	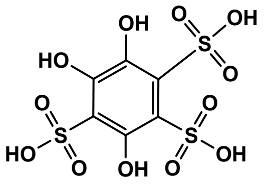	148.9551/298.917	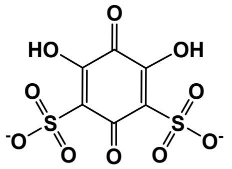 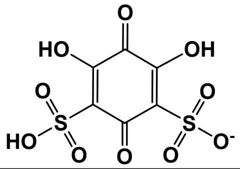
96.9603/194.931 /388.8915	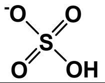	96.9604/194.9327	Same as left

Table S2 LC-MS Mass spectra of TAND samples and estimated molecule structures

Total ion chromatogram (Spectra extracted at blue and red dots)

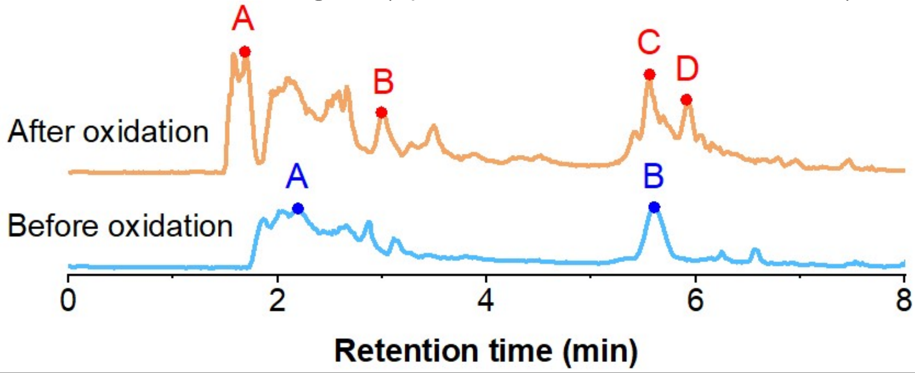

Before oxidation	After oxidation
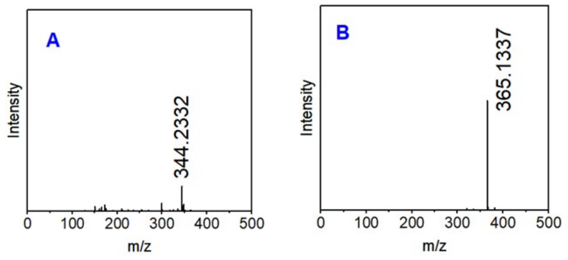	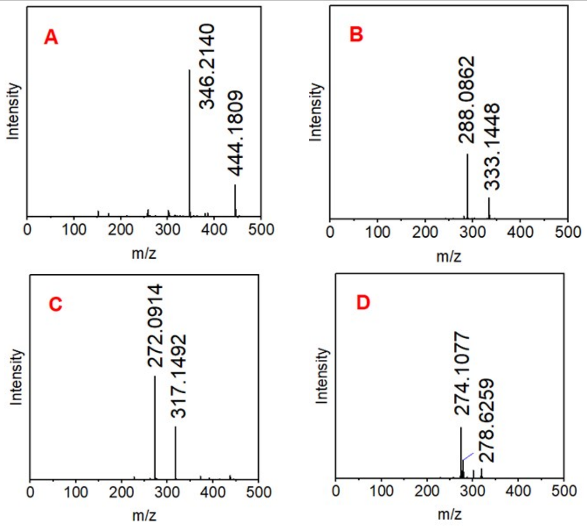

m/z	Estimated structure	m/z	Estimated structure
344.2332	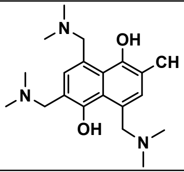	346.2140	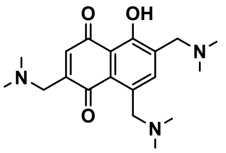
365.1337	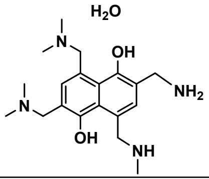	444.1809	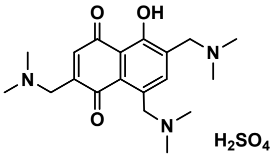
		288.0862	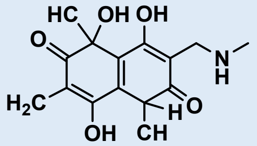
		333.1448	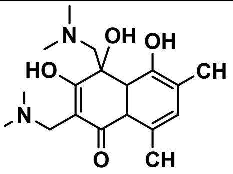
		272.0914	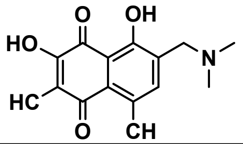

	317.1492	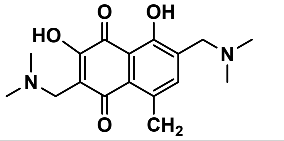
	274.1077	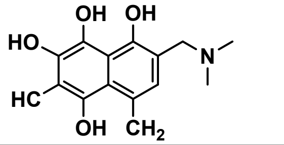
	278.6259	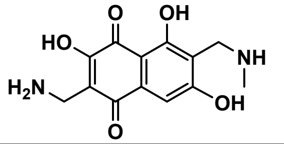
	304.0815<br>(spectra not shown above due to low signal)	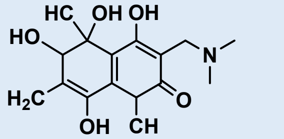
	302.1629<br>(spectra not shown above due to low signal)	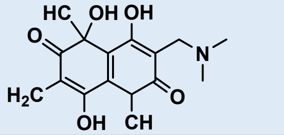

Reference

1. Yang, B.; Hoober-Burkhardt, L.; Krishnamoorthy, S.; Murali, A.; Prakash, G. K. S.; Narayanan, S. R., High-Performance Aqueous Organic Flow Battery with Quinone-Based Redox Couples at Both Electrodes. *Journal of The Electrochemical Society* **2016,** *163* (7), A1442-A1449.
